# Whose Perspectives Should Shape Progress? Community-Driven Recommendations for Person-Centered Upper Limb Difference Research

**DOI:** 10.21203/rs.3.rs-11460220/v1

**Published:** 2026-10-26

**Authors:** Susannah Engdahl, Alejandra Cano, Salena Drucker, Dena Foster, Katrina Kinsolving, Eric Nelson, Juliano Timm, John Yount, Cody McDonald, Siddhartha Sikdar

**Affiliations:** George Mason University; George Mason University; George Mason University; George Mason University; George Mason University; George Mason University; George Mason University; George Mason University; University of Washington; George Mason University

**Keywords:** amputation, community-engaged research, disability, limb difference, participatory research, prosthesis, rehabilitation, upper limb

## Abstract

**Objective:**

Research on upper limb prostheses often prioritizes technological advancement without integrating the lived experiences of people with upper limb difference into research goals and evaluation. A person-centered approach is needed to better align engineering efforts with the perspectives of those who are personally affected by the outcomes.

**Methods:**

We formed a team of seven individuals with upper limb difference and three research professionals to discuss pathways towards meaningful progress in prosthetics research. Conversations included topics like identity, societal views on disability, prosthesis usage patterns and preferences, and preferred terminology.

**Results:**

Community members emphasized the need for researchers to re-evaluate their assumptions about the true role of prostheses for people with upper limb difference. Using a collaborative, consensus-building process, we created four recommendations: 1) Focus research and translational priorities on supporting personal goals and abilities, not eliminating impairment, 2) The pursuit of long-term research and translational goals should be balanced with addressing more immediate community needs, 3) Research and clinical outcomes should be person-centered, not prosthesis-centered, and 4) Respect the unseen social-emotional impacts of living with upper limb difference.

**Conclusion:**

These recommendations can support equitable, person-centered research aligned with the priorities and lived experiences of people with upper limb difference.

## Background

Disuse of upper limb (UL) prostheses is frequently cited as a barrier to improving daily function among people with upper limb difference (ULD). Research and technological development aimed at addressing this problem have yielded increasingly sophisticated devices that mimic the articulated movements of an anatomical limb,^1^ as well as neuromuscular interfaces for more natural control and sensation capabilities.^2^ However, UL prosthesis abandonment rates have remained unchanged for decades^3,4^ despite these developments, suggesting that increased technical sophistication does not necessarily promote increased usage. Function is not solely related to prosthesis design given individual differences in usage patterns and/or desired outcomes based on biological, psychosocial, and environmental factors, underscoring the need for interventions tailored to the goals of people with ULD.^5,6^ It may also be debated whether abandonment is truly an appropriate metric for gauging the success of technologies or clinical care, as it assumes that prosthesis use is inherently valued by all people with ULD.

Central to these issues is a pervasive gap between the needs of people with ULD and the priorities of researchers working to provide solutions,^7^ potentially driven by technical development without a thorough understanding of end-user expectations.^4^ Surveys and qualitative methods can help bridge this gap but are often conducted at a single point in time, rely on structured questioning, and perpetuate researcher-participant power imbalances. Furthermore, these methods are often grounded in top-down investigator-developed research questions and hypotheses developed without input from people with ULD. As a result, these approaches may not fully capture the lived experience of ULD, limiting researchers’ ability to recognize and respond to nuanced, evolving community priorities.^8^

In contrast, community-engaged methods facilitate ongoing engagement so that stakeholders actively participate in research decision-making. Building on mutually respectful, beneficial, and cooperative partnerships between researchers and community members,^9^ outcomes can be improved through development of more appropriate interventions, broader dissemination of findings, and increased trust among marginalized groups.^10^ Community-engaged approaches in the context of ULD have been previously implemented through several co-design efforts (e.g., ^11,12^), but their use remains limited. Moreover, there have been few efforts to understand the holistic experience of ULD and how numerous physical, functional, and psychosocial factors influence prosthesis use.^13^

Here, we present recommendations developed through a community-engaged process to guide engineers, researchers, prosthetists, and other professionals involved in supporting people with ULD. These recommendations aim to better align research priorities and outcomes with the goals, preferences, and lived experiences of people with ULD.

## Methods

These procedures received an exempt determination from the Institutional Review Board at George Mason University (STUDY00000326). All community members provided written informed consent.

### Team Formation

Our team includes seven community members with ULD and three doctoral-level researchers working together as equal partners with complimentary strengths. The initial concept for our collaboration was initiated by two of the researchers, drawing on their professional backgrounds in wearable sensing for controlling prosthetic limbs, biomechanics, and functional outcome assessment. They subsequently invited community members from across the United States to participate. Community members were contacted due to their prior involvement in research studies led by the researchers or because they responded to online advertisements posted on forums/social media groups for individuals with limb difference. All community members were at least 18 years old, had congenital or acquired ULD, and either currently use or have previously used a UL prosthesis. The community members provided valuable expertise regarding the daily realities of ULD, gained through their personal lived experiences. Later, a third researcher was invited to provide complimentary expertise as a certified prosthetist-orthotist and an investigator who employs community-engagement techniques and qualitative methods to explore lived experiences and perceptions among people with limb loss or limb difference. Positionality statements from each author are provided in Appendix 1.

### Meeting Structure and Content

Our team met every month over Zoom for 17 months*. Meetings lasted one hour and covered topics such as personal identity and goal-setting, perception of disability, prosthesis use and related challenges, and scientific terminology. The meetings were conducted collaboratively to leverage the relative expertise of each team member. The researchers provided an initial discussion prompt based on their understanding of the field and insights shared by community members during prior meetings. Community members were invited to respond freely, with the researchers facilitating as needed. Conversations were allowed to evolve organically, giving community members a major role in choosing the direction.

### Writing Process

Meeting transcriptions were summarized by one of the researchers. Each meeting summary was presented to the community members at the subsequent meeting to allow clarification and promote a shared understanding. As the meetings progressed, we identified and refined the recurring primary themes to reflect consensus between all team members. While this was not an exhaustive or formal qualitative analysis, we followed a collaborative, iterative, consensus-building process involving both the researchers and community members.

Additionally, we selected first-person quotes from the meeting transcripts to support our conclusions. Quotes were lightly edited for clarity and brevity if needed (additional quotes are available in Appendix 2). We also chose to anonymize quotes to maintain team members’ privacy (CM = community member, RP = researcher/prosthetist; numeric identifiers do not indicate author order).

### Approach to Community Engagement

Community-engaged research exists on a spectrum with no single “correct” implementation,^14^ although the selected methods should ideally align with stakeholder interests to promote meaningful outcomes.^15^ Following guiding principles from the Patient-Centered Outcomes Research Institute,^16^ we pursued meaningful collaboration between researchers and community members by purposefully recruiting researchers and community members with varied backgrounds, paying community members after each meeting, seeking feedback from all involved parties to improve the partnership, regularly discussing the value of community input in research, and encouraging active participation in choosing discussion topics, preparing deliverables, and planning long-term objectives. These efforts helped ensure this work was truly cooperative and responsive to both researcher and community needs.

## Results

Across our discussions, community members emphasized a desire for genuine empathy toward their preferences and an appreciation for advances in prosthetic devices and patient care. Underlying these perspectives was a sense of self that sometimes included anger or grief but was largely driven by recognition that *“life is still beautiful and it’s worth living. Just because I’m missing a part of my body doesn’t make me any less whole than somebody that’s able-bodied”* (CM6). Many were reticent to identify as disabled *“because to myself, I still do all of the things”* (CM4) or preferred to not to dwell on their limitations since *“it doesn’t take very long and I’m very depressed. So when I start to go down that road, I quit”* (CM1). In fact, some viewed their ULD as a source of unique opportunities: *“I’m a world sailing champion. I worked for 30 years in a high-level government position. There’s a lot of people that have their arms and legs that had no chance to do any of that”* (CM2).

Many of our conversations explored the realistic role of prostheses for people with ULD, revealing that community members viewed them with varying degrees of importance. Some members extensively used their prostheses (e.g., *“I very seldom take my arms off. I wear them probably 18 hours a day, 7 days a week”* (CM1)), while others reported occasional use or no use at all (e.g., *“I rejected my two attempts at a prosthesis. They were not comfortable enough or useful enough to me functionally and I gave up”* (CM3)). However, frequency and duration of prosthesis use were not endorsed as primary indicators of a “successful” outcome. Our community members believed that a prosthesis may not be needed for every activity they want to do, and that difficulty using a prosthesis for specific activities they consider unimportant can be acceptable. Their judgements about prosthesis necessity were instead based on the specific task, context, and *“what expectations you have for yourself in your life. Like, I wasn’t going to go take the screwdriver and build something, so I’m not really bothered if I can’t do that. And so, when you think about it that way, that’s where everybody’s numbers, if you were to rate it, would probably be different.”* (CM4). Similarly, “perfect” task performance equivalent to that of someone without limb difference was not required because *“We cannot measure our success by an able-bodied person, what they can do. We measure our success by what we can do”* (CM1). As one member explained, *“In a sense, I’m only measuring against my own performance…If I want to eat my dinner conveniently, having my arm work and being able to pick my food up and put it in my mouth with my fork consistently…sometimes I drop the fork, and sometimes I drop the food, but I’m pretty happy with being able to feed myself”* (CM2). Other community members had goals that weren’t directly related to task performance, such as *“if I [can] wear it for whatever duration of time, and it’s comfortable. It doesn’t hurt my back. And this is probably different for other people, but just aesthetically there’s some outfits that I like the way it looks better whenever I am wearing my prosthetic. I know that’s just my own personal preference, but I feel like that could be a metric of how I measure the prosthetic”* (CM6). We also discussed how these personalized and varied views of our community members conflict with the “wildly different” (CM7) perspective of some researchers and clinicians. Specifically, this view expects prostheses to play a central role in daily functioning and treats able-bodied performance as the required standard, potentially overlooking the nuances of what people with ULD prioritize in their daily lives. As such, the researchers in our team called for a more flexible conceptualization based on a persons’ overall experiences, noting *“the current research literature really focuses on this more individual measure of success…we do want to talk about not only prosthesis use, but also when you’re not using your prosthesis. How does your conscious experience relate to your limb loss and your experience in the world?”* (RP3).

In addition to the role of the prosthesis, we extensively discussed what a prosthesis represents to our community members. Strikingly, most resisted viewing a prosthesis as a replacement for the absent limb (i.e., embodying the prosthesis). More often, they described a prosthesis as *“a tool to accomplish the things that need to be done, that I want to do. That is the main goal of having them, is to get things done, and to try to do things like I could do before. And to think of it as a limb, to me that’s preposterous”* (CM1). One of the members was more receptive to “replacing” the limb with a prosthesis but still had doubts: *“Maybe it’s because I’m younger, I could be biased. I feel like the sense of embodiment is something I kind of yearn for…[Prosthetics are] nice, they help me with my everyday life, but they’ll never be a part of me, and they’ll never replace my hand. And while I can agree with that, hopefully the benefit of using the prosthetic and being able to help in activities that you aren’t able to do without a prosthetic, that you’ll feel that sense of embodiment. But it won’t 100% be like, ‘This feels like my arm again.’ It’s kind of in a different way”* (CM6). Ultimately, they were pragmatic about their perceptions of prostheses, repeatedly emphasizing *“the reality that it’s a tool for me. Sure, I would like to have a better tool. But I don’t think it will ever become a replacement for the arm that I lost”* (CM5). They were instead focused on more tangible aspirations, like finding *“what can help me get what I need done and in the easiest, most natural way. I don’t believe I ever expected that to feel like it was part of my body* (CM4)*”.* In fact, *“just because it doesn’t feel like it’s part of me or an extended part of my limb or something, it’s not deterring me from wanting to use it or just giving up on prosthetics altogether”* (CM6). One member even felt that viewing the prosthesis as a replacement limb may be harmful, whereas *“the healthier thing [is] to say that I accept my body as it is now, and now I’m looking for tools in order to focus on living it the best that I can”* (CM7).

To better understand our community members’ aversion to embodiment, we debated alternative viewpoints, including “*one argument that a researcher might make is that the [prosthesis] function isn’t there because the embodiment isn’t there, and so that’s why we should focus on embodiment”* (RP2). This proposition proved to be unconvincing, as one member immediately quipped in response: *“That sounds like a two-handed person said that”* (CM3). Ultimately, the members agreed that while they could appreciate researchers’ well-intentioned enthusiasm, it was *“ridiculous to think that something could replace a natural limb. And I’m gonna go a little hard here. I think that there is a little bit of scientific ego that gets in the way with that, and that’s where the push for embodiment comes in. A Frankensteinian fetish, if you will, that there is an ability, that there is a way to fully replace a natural limb”* (CM7). Moreover, they speculated that *“the whole idea of embodiment is maybe the people that are developing and studying need a goal, and they came up with a word for what they want it to be. Primarily being people who probably have their limbs, they’re probably also trying to figure out if they were in this situation, what they would want out of their work, and what they’re developing, and what their highest goal would be” (CM4)*. Members believed developers may misunderstand the lived experience of ULD, and argued “*if we’re not being talked to, if we’re not being discussed for what it is we’re actually looking for, it’s the most absurd process in the world”* (CM7). This opinion was echoed by one of the researchers who shared, *“As a prosthetist, I would agree with you. I think [embodiment is] very much a researcher term”* (RP1). Given that these views may be overlooked, the same researcher invited greater consideration of *“How can [developers] bring prosthetists and the people who will potentially be using this technology down the road, along for the journey? To me, that’s as big a goal as the technology itself”* (RP1).

Nonetheless, community members stressed that they were not *“against prosthetic development at all… More research can lead to something better that will suit you”* (CM5). They were grateful that what currently exists is “*fantastic compared to what we had [40 years ago]…They have made some really extraordinary mechanical and material innovations”* (CM2). Further practical improvements were still strongly desired, even if it doesn’t lead to the prosthesis becoming “part of” them. Members believed the essential qualities of a prosthesis were to *“function well, be comfortable, easy to use, but also durable”* (CM5). When a prosthesis falls short of these expectations, it leads to *“people rejecting their prosthetics all the time. If they have one hand, they say, ‘Hey, I can do everything with one hand, and I’m not going to be uncomfortable, and wear this thing that hurts, and doesn’t really make a lot of difference in my life’”* (CM2). Members maintained a practical outlook, acknowledging that *“If it was a real arm, that would be so much better, but it’s not going to be. I do try to keep kind of that perspective of it is a mechanical implement…I think the part where we would like it to be a little bit better might be more focused on, if it doesn’t hinder me while it helps me”* (CM4). Pursuing smaller technical development steps towards larger goals was suggested as a way to accomplish this. One member explained *“I would first want the interface. I would want something that is reliable, even if it only had, like, 3 functions first, to know that what I say goes, and it’s going to act reliably every single time…once you get that interface really solid, then you can still continue to work on another prosthetic model that can do more functions”* (CM7). Some members also valued device simplicity, seeing *“no reason to have all this extra stuff that I have to worry about breaking down and everything. I’m very simple in that way”* (CM1). Members also noted concerns about accessibility and cost, as these factors are not always within the user’s control: *“it’s also what insurance can pay [for], right? I’m afraid of using my bebionic and damaging it. I don’t know for how long my insurance will make repairs…Definitely, we need better technology. There is no question. But it has to be also accessible to everyone”* (CM5).

Even with improved prostheses, our community members felt their accomplishments were primarily attributable to their own effort and determination. A person may *“have all the tools in the books, the defenses, prosthetics, or whatever. But if you don’t have the will to go out, to do things, you don’t do it”* (CM5). This view emphasizes personal agency, with the prosthesis serving as a means for exercising that agency. In fact, some did not feel that ULD or prosthesis use uniquely limited their agency. One member explained that *“all people, as we age, are getting used to losing some agency a little bit at a time…disability becomes less of a distinguishing feature as you get old because everybody else is slipping as well”* (CM3). Members cautioned that overemphasizing the technology itself conflicts with their perception of prostheses as “tools for personal agency” by obscuring the person who the technology is designed to support. This conflict can manifest in the way prostheses are publicly presented. One member illustrated this concern using the breast cancer awareness slogan “Save the Tatas,” which has received pushback for being *“focused on specifically just saving breasts…instead of saying, ‘Save women, save women’s lives.’ And so I think that there has to be a shift in ‘Yes, we want prosthetic development, but it still needs to be very, very person-centered’”* (CM7). In the UL prosthesis space, a similar dynamic occurs with *“some of the [device] names, like the Hero Arm, the Zeus, the this and that. Take [the ego] down a little bit and make sure that we see that it’s the clients that we’re really focusing on, and not so much the incredible nature of the prosthetic. That the clients retain the power of agency, and that prosthetics remain a tool of that agency”* (CM7).

Ultimately, differing beliefs about ULD the role of UL prostheses can lead “*engineers [to] feel a grief or a loss for [people with ULD], but it almost functions like toxic positivity instead of realism like, ‘Let’s accept what’s real but still get you to a really good position’*” (CM7). This disconnect may create an expectation that people with ULD should continually look for and experiment with new devices as technology improves, but our community members described varying levels of comfort around this. For example, one member actively keeps up with engineering advancements due to being *“obsessed with prosthetics…my newsfeed turns up all these articles about prosthetics”* (CM2). In contrast, another member had participated in a prosthetics research study and described internalizing the researchers’ feelings, where *“if they can’t get the sensors to work or whatever, I just feel so bad. I feel bad for them. And I’m like, is it my fault that I do this? I could feel their sadness and hope just deflating in the room”* (CM7). These negative emotions resulting from engaging with developers and/or participating in research are rarely considered and yet may influence some people with ULD to avoid research studies.

Similar dynamics can occur in clinical care, where people with ULD may perceive their prosthetists’ frustration with an unsuccessful outcome as being directed toward them. One community member recalled an interaction that *“was so embarrassing for these guys who were supposed to be the best [prosthetists] there are, and they spent so much time and energy making these prosthetics. When [the prosthetics] didn’t work, I don’t know if I felt worse for [the prosthetists] than I did for myself…it’s like they couldn’t do it at all, and they ended up blaming me”* (CM2). These tensions may arise due to how inextricably linked prosthetists are to the devices they provide. Additionally, prosthetists are in positions of power, and people with ULD “*rely a lot on the prosthetist to tell you what’s good for you, but it’s a lot his or her opinion…We are very dependent on one person, the prosthetist. It’s a very unequal relationship”* (CM5). Without active consideration of this power dynamic, prosthetists may inadvertently contribute to unrealistic patient expectations. For example, when one member’s new prosthetic suspension and socket design didn’t work after repeated clinical visits, they were *“very disappointed and I’m pretty sure I went home and cried…that could have been prevented if [the prosthetist] had been a little bit more transparent of what the outcome would be, and not what he or I wanted it to be”* (CM6). Ultimately, community members desired greater empathy and consideration of financial and emotional costs associated with trying new technology or seeking care: *“It’s so hard to get the new [prosthetic], right? Whether it’s I have to qualify for it, I have to do the insurance stuff, or it’s really expensive, or I’m going to have to practice for at least a year or something with the new device to even see if this is going to be better and more functional for me. And so, there’s this incredible ask to try something new on so many different levels and I think that also builds in a little bit of a reticence to try the new things… It is devastating to put that much energy, time, money, hope, which is the most valuable of all resources, into something new and not have it pan out”* (CM7).

## Discussion

As a team, we believe that people with ULD have extensive insights that should inform future research priorities and considerations. Our community members were grateful for ongoing research, but also stressed that their lived experiences, priorities, and preferences must be meaningfully integrated. A recurring theme was the need to reconsider assumptions about the role of prosthetic limbs, thereby aligning research priorities with what people with ULD identify as most meaningful rather than with purely technology-driven objectives. Together, these insights point to the importance of person-centered research that responds to both shared community priorities and individual needs.

Based on our discussions, we offer four recommendations to support the active integration of community perspectives in guiding research goals, language, and procedures: 1) *Focus research and translational priorities on supporting personal goals and abilities, not eliminating impairment*, 2) *The pursuit of long-term research and translational goals should be balanced with addressing more immediate community needs*, 3) *Research and clinical outcomes should be person-centered, not prosthesis-centered*, and 4) *Respect the unseen social-emotional impacts of living with ULD*. These interconnected recommendations relate to how objectives are framed, success is evaluated, and different perspectives are integrated in pursuit of common goals in research and clinical settings.

Our recommendations point to several ways that researchers can translate community perspectives into practice. One approach is to articulate research motivations (e.g., in manuscripts or proposals) around supporting the lives that people with ULD want to lead, rather than presenting ULD as a problem to be solved. Descriptions of the “impairment”, “suffering”, or “devastating consequences” of ULD (e.g., ^3,17,18^) may unintentionally propagate a deficit-oriented narrative in which researchers are the primary agents of change and people with ULD are passive recipients. Our community members acknowledged experiencing some challenges but also emphasized their ability to pursue many of the activities they value. Having the right tool for the job is paramount to success in accomplishing those goals, but that “tool” may be a prosthesis, residual limb, contralateral arm, foot, assistive device, or another person’s help. While a prosthesis may be essential for some, a prosthesis isn’t always necessary for a person to feel capable and independent while pursuing their desired activities.^19^ Thus, researchers should consider how to support the range of strategies people with ULD use to achieve their objectives, rather than focusing solely on prostheses.

Relatedly, each individual with ULD has a different set of interests and priorities in tasks for their prosthetic device to help them complete. If the full range of possible task needs are not considered by researchers while developing components and prosthetists while providing care, the user is less likely to be happy with their device and also less likely to wear it. Thus, advancements should not be measured solely by improvements in technology or physical function. Progress may also be evaluated by how well research supports the goals, abilities, and lived experiences of people with ULD. This broader view of progress places the person, not the prosthesis, at the center of research objectives.

While many studies have focused on outcome measurement, the selected metrics often encompass only a portion of the International Classification of Functioning, Disability and Health (ICF), which describes disability as an interaction between bodily functions/structures, activities, participation, and environmental factors.^20^ Prosthesis-centered research tends to emphasize the first two domains through studies of prosthesis design^21^ and task-based assessments of dexterity and function.^21–23^ However, these metrics may not capture the full spectrum of “function” that people with ULD desire. Because “function” is context- and person-dependent, it may need to be redefined and measured in ways that capture this temporal and individual variation. As noted by Caldas et al.,^13^ functional outcomes are highly interrelated with prosthesis features, as well as psychological and contextual considerations.

Person-centered research does not diminish the value of technical innovation; rather, it seeks to align different perspectives so researchers do not overemphasize areas that are unimportant to community members or underemphasize priorities that are. While some of this divergence may stem from lack of communication among stakeholders, it may also be propagated by systemic issues. For example, programmatic priorities at funding agencies often reward novelty, potentially disincentivizing researchers from pursuing work that may be perceived as incremental even if it addresses urgent community needs. Another systemic issue is the broader societal assumption that ULD is a personal deficit in need of correction. This belief positions the prosthesis as the default solution to ULD, rather than as one option among many available approaches. It may also influence professional identity and training for both researchers and clinicians, whose expertise often centers on prosthetic devices as the ideal solution for ULD. The expected importance of prostheses also seems to be reflected in definitions used by U.S. health insurance providers (e.g., ^24^) and regulatory agencies (e.g., ^25,26^), which describe the prosthesis as a replacement for missing anatomical structures and functions. While it is understandable that clear definitions are necessary for policy and regulatory frameworks, they may contribute to an oversized emphasis on prostheses in research and clinical settings. We acknowledge that addressing systemic influences is complex and do not believe that doing so should come at the expense of technological progress; both the researchers and community members on our team advocate for continued efforts towards developing more advanced devices. Accordingly, we suggest a balanced approach that pursues innovation while also remaining responsive to people’s immediate needs and priorities.

Our discussions generated some concrete examples of between research priorities and community priorities, particularly regarding embodiment (i.e., perception of the prosthesis as “part of” the body rather than a “tool”) and socket comfort. First, although researchers have increasingly identified embodiment as a crucial topic of study, little evidence suggests that it is necessary for prosthesis acceptance among people with ULD.^27^ Similarly, our community members repeatedly stated that they prefer to view their prosthetic device as a tool rather than an extension of their body, noting they were less concerned with replacing their altered limb and more interested in the function, durability, comfort, and reliability of their prosthesis. Users would like to be able to rely on their prosthetic device with unquestionable faith that they will be able to complete their daily tasks without concern for the prosthesis. It should work as needed, every time, for each task. In contrast, our community members often mentioned socket comfort as a key to long-term prosthesis use. They stressed that the device must fit without causing pain, discomfort, or impeding the movement of the limb. If the prosthesis causes more issues than not wearing it, the user is not likely to put the effort into wearing, learning, and acclimating to the device. Yet, despite the importance of socket comfort to people with ULD, it remains understudied in the literature.^28^ Together, these examples illustrate the importance of considering community needs and preferences when setting research agendas: researchers developing UL prostheses would be more successful in the eyes of the users by focusing on practical improvements in function, durability, and comfort while using a person-centered approach.

Community members also described challenges of living with ULD that extend beyond physical function and prosthesis use to include less visible emotional and social experiences. For researchers, it is important to recognize that these experiences can shape how people with ULD define success, interact with others, and make decisions about prosthesis use. While not every study needs to examine psychological or social outcomes, person-centered research should consider how these experiences may influence prosthesis design priorities and evaluation metrics. Unintended emotional impacts can also occur in both clinical and research settings. For example, if realistic outcomes are not clearly explained during clinical care, patients and prosthetists may feel disappointment and frustration, especially when considerable time and/or resources have been invested. Similarly, during laboratory testing of non-commercial devices, study subjects may devote substantial time and effort to participating. If the experiment “fails”, participants may interpret the outcome as a personal failure or feel culpable for circumstances beyond their control, like technical problems with the prototype. In both cases, the person with ULD may feel responsible for managing their own reactions, as well as that of the prosthetist or researchers. Clear, transparent communication about expectations, limitations, and outcomes can help prevent these situations and ensure that emotional impacts do not fall disproportionately on the person with ULD.

Our recommendations were collaboratively determined through a consensus-driven process rooted in community members’ expertise and lived experience. To strengthen the rigor of this inherently subjective approach, our team was deliberately chosen to include community members with diverse demographic characteristics and limb difference etiologies and researchers from varied professional backgrounds. We also conducted monthly real-time checks to review and clarify summaries of past discussions. This was not a formal qualitative analysis, but rather a collaborative co-creation of knowledge through ongoing discussion and reflection. As such, readers are encouraged to critically consider transferability of our recommendations. Nonetheless, we believe these co-created perspectives offer valuable insights that may be overlooked with traditional study designs that favor large, homogeneous groups or avoid disaggregation of subgroups.^29^ Incorporating the “expert opinions” of community members into the literature, similar to how clinician expertise is used to inform clinical practice guidelines (e.g., ^30^), is an important alternative paradigm.

## Conclusion

Our discussions revealed that research priorities should be better aligned with the diverse and nuanced realities of living with ULD. This alignment may support person-centered approaches that more fully accommodate individual preferences, goals, and lived experiences. Incorporating community perspectives into research priorities, technology development, and outcome evaluation can help ensure future work is meaningful to people with ULD.

## Supplementary Material

This is a list of supplementary files associated with this preprint. Click to download.

• Appendix1.pdf

• Appendix2.xlsx

## Data Availability

Data sharing not applicable to this article as no datasets were generated or analyzed during the current study.
